# Cell Death Mechanisms Induced by CLytA-DAAO Chimeric Enzyme in Human Tumor Cell Lines

**DOI:** 10.3390/ijms21228522

**Published:** 2020-11-12

**Authors:** María Fuentes-Baile, Pilar García-Morales, Elizabeth Pérez-Valenciano, María P. Ventero, Jesús M. Sanz, Camino de Juan Romero, Víctor M. Barberá, Cristina Alenda, Miguel Saceda

**Affiliations:** 1 Unidad de Investigación, Fundación para el Fomento de la Investigación Sanitaria y Biomédica de la Comunidad Valenciana (FISABIO), Hospital General Universitario de Elche, Camí de l’Almazara, 11, 03203 Elche (Alicante), Spain; mariafuentesbaile@gmail.com (M.F.-B.); camino.dejuan@gmail.com (C.d.J.R.); barbera_vicjua@gva.es (V.M.B.); 2 Instituto de Investigación, Desarrollo e Innovación en Biotecnología Sanitaria de Elche (IDiBE), Universidad Miguel Hernández, Avda. Universidad s/n, Ed. Torregaitán, 03202 Elche (Alicante), Spain; pgarcia@umh.es (P.G.-M.); elizabethpv2908@gmail.com (E.P.-V.); 3 Unidad de Investigación, Instituto de Investigación Sanitaria y Biomédica de Alicante (ISABIAL), Hospital General Universitario de Alicante, C/Maestro Alonso, 10, 03010 Alicante, Spain; maripazvm@gmail.com (M.P.V.); alenda.cris@gmail.com (C.A.); 4 Centro de Investigaciones Biológicas Margarita Salas (Consejo Superior de Investigaciones Científicas) and Centro de Investigación Biomédica en Red de Enfermedades Respiratorias (CIBERES), C/Ramiro de Maeztu, 9, 28040 Madrid, Spain; jmsanz@cib.csic.es; 5 Unidad de Genética Molecular, Hospital General Universitario de Elche, Camí de l’Almazara, 11, 03203 Elche (Alicante), Spain

**Keywords:** cancer therapy, reactive oxygen species, oxidative damage, mitochondrial membrane potential, calcium mobilization, PARP-1, AIF, apoptosis, necrotic-like cell death

## Abstract

The combination of the choline binding domain of the amidase N-acetylmuramoyl-L-alanine (CLytA)-D-amino acid oxidase (DAAO) (CLytA-DAAO) and D-Alanine induces cell death in several pancreatic and colorectal carcinoma and glioblastoma cell lines. In glioblastoma cell lines, CLytA-DAAO-induced cell death was inhibited by a pan-caspase inhibitor, suggesting a classical apoptotic cell death. Meanwhile, the cell death induced in pancreatic and colon carcinoma cell lines is some type of programmed necrosis. In this article, we studied the mechanisms that trigger CLytA-DAAO-induced cell death in pancreatic and colorectal carcinoma and glioblastoma cell lines and we acquire a further insight into the necrotic cell death induced in pancreatic and colorectal carcinoma cell lines. We have analyzed the intracellular calcium mobilization, mitochondrial membrane potential, PARP-1 participation and AIF translocation. Although the mitochondrial membrane depolarization plays a crucial role, our results suggest that CLytA-DAAO-induced cell death is context dependent. We have previously detected pancreatic and colorectal carcinoma cell lines (Hs766T and HT-29, respectively) that were resistant to CLytA-DAAO-induced cell death. In this study, we have examined the putative mechanism underlying the resistance in these cell lines, evaluating both detoxification mechanisms and the inflammatory and survival responses. Overall, our results provide a better understanding on the cell death mechanism induced by CLytA-DAAO, a promising therapy against cancer.

## 1. Introduction

Cell death is a necessary phenomenon for the normal development of vertebrate and invertebrate organisms. Through this process, organisms discard damaged cells in pathological conditions such as ischemic diseases, viral infections and radiation damage, among others. A few years ago, it was only possible to differentiate between two forms of death: apoptosis and necrosis [1]. In recent years, researchers have extensively studied the different mechanisms by which cells die in response to different stimuli [2,3,4] allowing the development of a wide range of well-characterized cell death mechanisms. The main forms of cell death have been divided into subclasses, as variations in the mechanism have been described, providing a better understanding of how this phenomenon works. Although all forms of cell death have different morphological and molecular characteristics, they are closely related and, in many cases, interconnected.

The combination of resistance to programmed cell death and genetic instability, two hallmarks of tumor cells, often leads to chemo- and radio-resistance [5]. Therefore, acquiring insights into cell death and drug resistance mechanisms is crucial for the development of effective treatments against cancer.

A growing strategy for cancer treatment is the so called suicide therapy, which consists of the use of genes or enzymes that transform a non-toxic prodrug in a toxic compound causing tumor cell death [6]. Delivering these enzymes, which are harmless by themselves, specifically to the tumor area, increases effectiveness and abolishes secondary effects related to classical anticancer therapies [7]. Recently, we have evaluated the effect of the D-amino acid oxidase (DAAO) protein from *Rhodotorula gracilis*, which catalyzes the oxidation of D-amino acids and generates hydrogen peroxide as a by-product in tumor cell lines [8]. Furthermore, we use a chimera formed by the DAAO enzyme bound to the choline-binding domain of the amidase N-acetylmuramoyl-L-alanine (CLytA) from *Streptococcus pneumoniae* [9]. Thus, the CLytA-DAAO chimeric protein can be immobilized on nanoparticles that contain choline or analogues such as diethylaminoethanol (DEAE) on their surface which opens the possibility of converting DAAO treatment in targeted therapy [10].

The combination of CLytA-DAAO with D-Ala, either free or immobilized in magnetic nanoparticles, induces cell death on several pancreatic and colorectal carcinomas and in glioblastoma cell lines [11]. CLytA-DAAO generates oxidative stress, causing irreparable DNA damage and ultimately leading to cell death. The blockade of CLytA-DAAO-induced cell death by a pan-caspase inhibitor in glioblastoma cell lines suggests that a classical apoptosis mechanism occurs. However, the cell death induced by the same treatment in the rest of pancreatic and colorectal carcinoma cell lines was not inhibited by caspase inhibitors, necrostatin-1, chloroquine, spautine-1 or ferrostatin-1 [11]. In this way, other forms of cell death such as necroptosis, autophagy and ferroptosis were discarded as mechanisms implicated in this process. Accordingly, a putative mechanism was proposed regarding pancreatic and colorectal carcinoma cell lines—i.e., that CLytA-DAAO induces a necrotic-like cell death.

Our laboratory has identified several cell lines totally or partially resistant to CLytA-DAAO-induced cell death. These cell lines presented different profiles in terms of free radical generation and DNA damage, indicating the existence of different resistance mechanisms. In some cell lines, the resistance mechanisms appear to be related to the detoxification of free radicals generated by the CLytA-DAAO-catalyzed reaction, while in others it would depend on signaling pathways related to cell survival and damage repair [11].

In the current study, we tested parthanatos as a possible mechanism involved in the cell death induced by CLytA-DAAO in pancreatic and colorectal carcinoma cell lines. Parthanatos is well-defined as a necrosis-like cell death that occurs when there is excessive DNA damage and has been widely associated with drugs that generate oxidative stress [12,13]. This form of cell death is characterized by the overexpression of Poly (ADP-ribose) polymerase 1 (PARP-1), the cytosolic accumulation of polymeric PAR, the translocation of apoptosis-inducing factor (AIF) from the mitochondria to the nucleus, the mobilization of intracellular calcium and the depolarization of the mitochondrial membrane [14].

We found that CLytA-DAAO induces apoptotic and different types of mitochondrial permeability transition (MPT)-driven necrosis (due to the opening of permeability transition pores in the mitochondria) and even parthanatos cell death, depending on the cellular context. These data together with the lack of effect in non-tumoral cells, that we have previously described [11], suggest that CLytA-DAAO treatment could be widely use.

Finally, we have analyzed the resistance mechanisms against CLytA-DAAO-induced cell death that are present in Hs766T, a pancreatic carcinoma cell line, and in HT-29, a colorectal carcinoma cell line. We have described the role in this process of several genes and transcription factors involved in the inflammatory response, cell survival and detoxification. Our results suggest that the resistance mechanisms are pleiotropic in all the cell lines tested, although we have identified several common interrelated signaling pathways that allow these cell lines to avoid CLytA-DAAO-induced cell death.

## 2. Results

### 2.1. Calcium Release from Cellular Reservoirs Is Responsible for the MMP Decrease That Leads to Cell Death in Most of the Cell Lines after CLytA-DAAO Treatment

**Figure 1:**
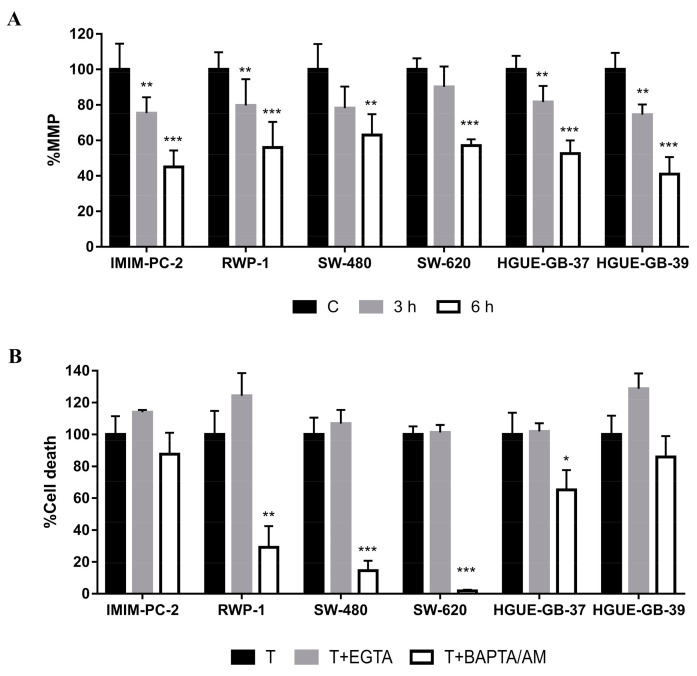
Role of mitochondrial membrane potential (MMP) and calcium in CLytA-DAAO-induced cell death. (**A**) IMIM-PC-2, RWP-1, SW-480, SW-620, HGUE-GB-37 and HGUE-GB-39 cell lines were treated with CLytA-DAAO and D-Ala for 3 or 6 h and the MMP was determined using MitroTracker Green FM and MitoTracker Red CMXRos fluorescent dyes. Data represent the percentage of MMP, normalized with the control cells (Cs) as 100% ± SD with n ≥ 6. (**B**) IMIM-PC-2, RWP-1, SW-480, SW-620, HGUE-GB-37 and HGUE-GB-39 cell lines were treated with CLytA-DAAO and D-Ala (T) in the presence or absence of BAPTA/AM or EGTA as described in the materials and methods section and cell death was determined by flow cytometry. Data represent the percentage of cell death, normalizing the treatment with CLytA-DAAO as 100% ± SD with n ≥ 3. * indicates a *p*-value < 0.05, ** *p*-value < 0.01 and *** *p*-value < 0.001.

In order to gain insights into the mechanism of CLytA-DAAO-induced cell death, we analyzed the mitochondrial membrane potential (MMP) after CLytA-DAAO treatment. Mitochondrial membrane depolarization was found in all cell lines tested during the first hours of treatment with CLytA-DAAO (Figure 1A).

To examine whether the entry of calcium into the mitochondria could be responsible for an MMP decrease, we analyzed the role of calcium in CLytA-DAO-induced cell death. We pretreated cells with two calcium chelators in order to inhibit extracellular calcium uptake (EGTA) or intracellular calcium release from cellular reservoirs (BAPTA/AM). CLytA-DAAO-induced cell death was blocked upon BAPTA/AM treatment whereas cells pretreated with EGTA did not show a decrease in cell death, confirming the importance of intracellular calcium release in this process. Interestingly, this effect was not only present in the pancreatic and colorectal carcinoma cell lines, but also in glioblastoma cell lines (Figure 1B). However, some tumor cell lines such as the IMIM-PC-2 (pancreatic carcinoma) and HGUE-GB-39 (glioblastoma) cell lines did not respond to these treatments with a significant change in cell death. Together, these findings suggest that calcium mobilization inside the cell is essential for the cell death effect shown in most cell lines. Moreover, the results observed in IMIM-PC-2 and HGUE-GB-39 indicate the existence of other mechanisms involved in the mitochondria membrane depolarization that does not include intracellular calcium mobilization and its possible entry into the mitochondria.

Regarding glioblastoma cell lines, the movement of calcium from the ER to the mitochondria, and the consequent lowering of the MMP, is one of the events that usually occurs in the apoptotic process [15]. Our results with the MMP and calcium mobilization led us to hypothesize that the CLytA-DAAO-induced cell death in pancreatic and colorectal cell lines could be parthanatos, a necrosis-like cell death related to oxidative stress and DNA damage, in which intracellular calcium mobilization associated with a dissipation of the mitochondrial membrane potential occurs [13,16].

### 2.2. CLytA-DAAO Induces Different Cell Death Mechanisms Depending on the Carcinoma Cell Lines

**Figure 2:**
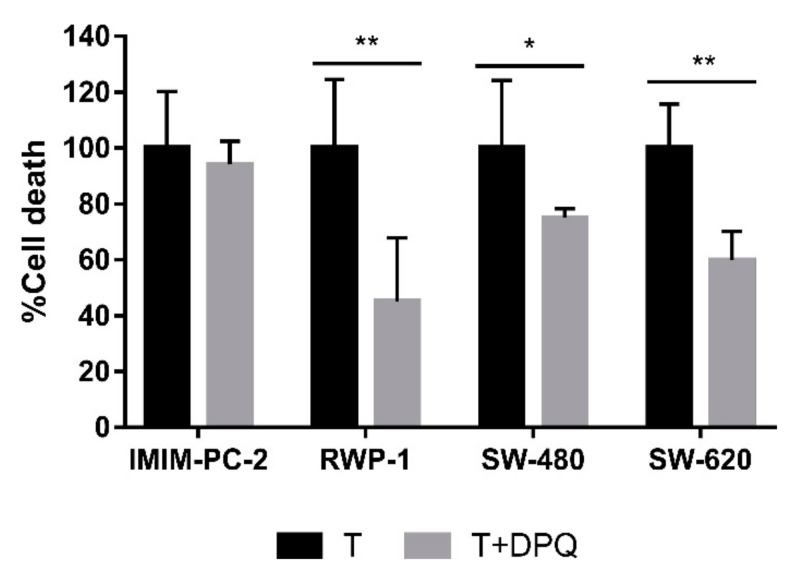
Effect of PARP-1 inhibition in CLytA-DAAO-induced cell death. IMIM-PC-2, RWP-1, SW-480 and SW-620 cell lines were treated with CLytA-DAAO and D-Ala (T), in the presence or absence of 3,4-Dihydro-5(4-(1-piperindinyl)butoxy)-1(2H)-isoquinoline (DPQ) for 24 h and cell viability was determined by flow cytometry. Data represent the percentage of cell death, normalizing the treatment with CLytA-DAAO as 100% ± SD with n ≥ 3. * indicates a *p*-value < 0.05 and ** *p*-value < 0.01.

Because PARP-1 mediates parthanatos when it is overactivated, we investigated the role of PARP-1 in CLytA-DAAO-induced cell death, pretreating pancreatic and colorectal carcinoma cell lines with 3,4-Dihydro-5(4-(1-piperindinyl)butoxy)-1(2H)-isoquinoline (DPQ), a PARP inhibitor [17,18]. PARP-1 has a double role in the DNA damage response; it is involved in the DNA base-excision repair system and it also promotes parthanatos cell death when intense DNA damage exists [14]. Our previous observations in glioblastoma cell lines indicate that PARP-1 inhibition potentiates CLytA-DAAO-induced cell death [11]. After inhibiting PARP-1, CLytA-DAAO-induced cell death decreased in all the pancreatic and colorectal carcinoma cell lines tested, with the exception of the IMIM-PC-2 pancreatic carcinoma cell line (Figure 2), suggesting that PARP-1 plays a role in CLytA-DAAO-induced cell death in most of the cell lines tested.

AIF exits from the mitochondria to cytosol and its subsequent entry into the nucleus is a characteristic process that occurs both in apoptosis [15,19] and parthanatos cell death [20,21]. Next, we sought to understand whether CLytA-DAAO-induced cell death involves AIF translocation in our three cancer models. To determine the AIF location before and after the CLytA-DAAO treatment, immunocytochemistry was performed using a specific AIF antibody and cells were visualized by confocal microscopy. Consistent with our previous studies, our analyses after CLytA-DAAO treatment revealed AIF translocation from mitochondria to the nucleus mainly in HGUE-GB-37 and HGUE-GB-39 glioblastoma cell lines as expected in apoptotic cell death. Among pancreatic and colorectal carcinoma cell lines, AIF translocation to the nucleus was detected predominantly in RWP-1, followed by SW-620, and was hardly observed in SW-480 and IMIM-PC-2. To validate these observations, we performed densitometry measurements where the green fluorescence intensity in the nucleus was quantified (Figure S1).

**Figure 3:**
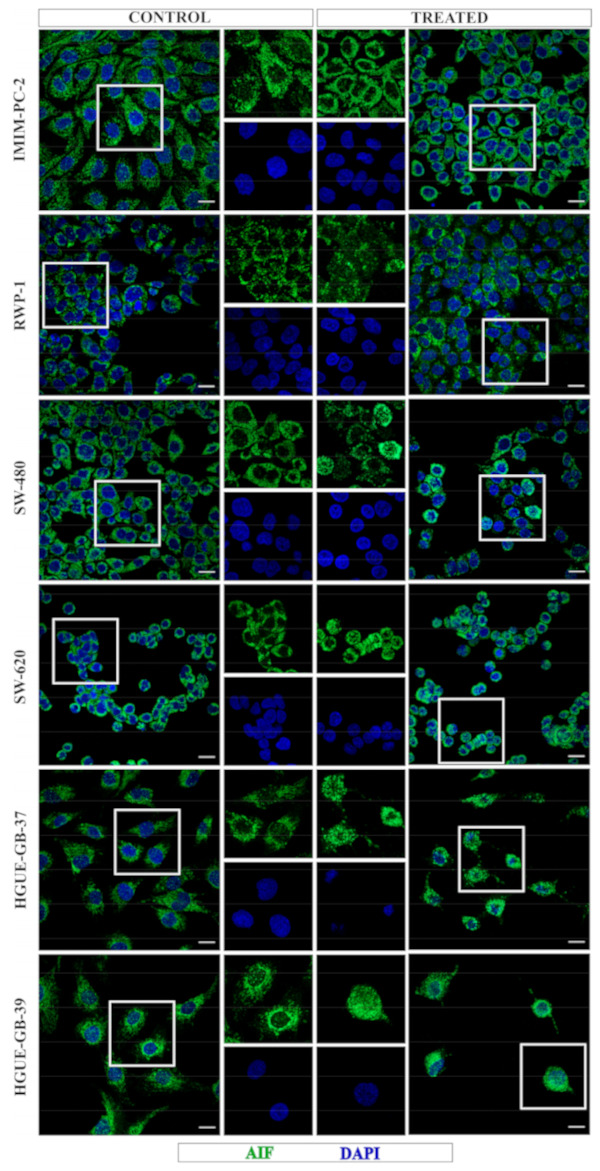
AIF translocation during CLytA-DAAO-induced cell death. IMIM-PC-2, RWP-1, SW-480, SW-620, HGUE-GB-37 and HGUE-GB-39 cell lines were untreated (C) and treated (T) with CLytA-DAAO and D-Ala for 6 h. Images show AIF (green) and 4′,6-diamidino-2-phenylindole (DAPI) (blue) staining. Central images display an amplification of the white box. Scale bar, 20 μm.

Remarkably, the nuclear AIF levels were much lower in all cell lines as compared with those observed in glioblastoma cell lines. Furthermore, cell lines with low AIF nuclear translocation presented an accumulation in the perinuclear region (Figure 3). It is possible that a different time-course after treatment may be necessary to detect a significant AIF entry into the nucleus in pancreatic and colorectal carcinoma cell lines, or maybe AIF accumulates in the perinuclear region colocalized with chromatin, as has been observed in some types of programmed cell death [22,23,24]. Altogether, these results strongly suggest that CLytA-DAAO-induced cell death is probably parthanatos in the RWP-1 cell line, while in the IMIM-PC-2, SW-480 and SW-620 cell lines the role of AIF requires further analysis.

### 2.3. MAPKs Pathways Are Involved in CLytA-DAAO-Induced Cell Death

**Figure 4:**
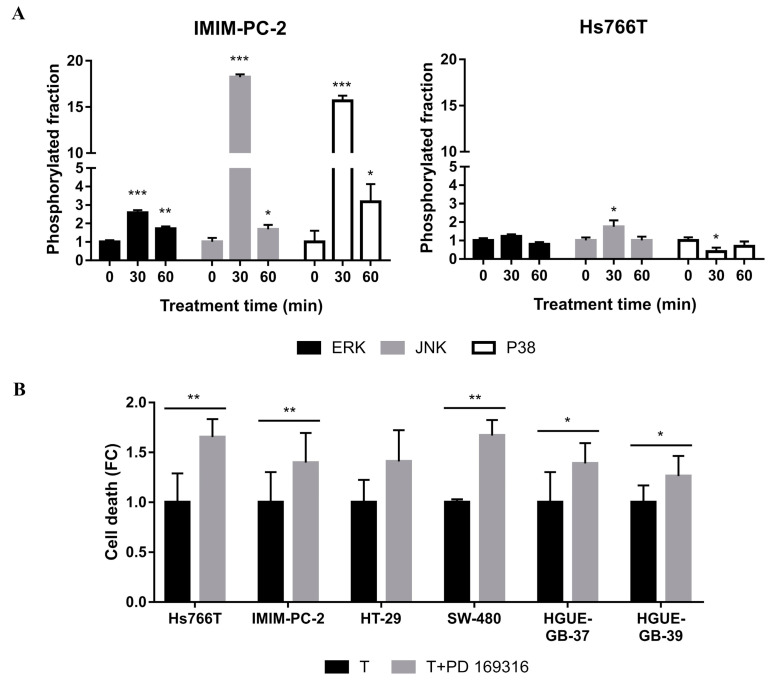
MAPK implication in CLytA-DAAO-induced cell death. (**A**) IMIM-PC-2 and Hs766T cell lines were treated with CLytA-DAAO and D-Ala for 30 min and 1 h and ERK, JNK and P38 phosphorylation was determined by ELISA assay. Data represent the fraction of MAPK phosphorylation, normalizing the phosphorylation in control cells as 1 ± SD with n ≥ 3. (**B**) Hs766T, IMIM-PC-2, HT-29, SW-480, HGUE-GB-37 and HGUE-GB-39 cell lines were treated with CLytA-DAAO and D-Ala (T) in the presence or absence of PD 169316, a p38 inhibitor, for 24 h and cell viability was determined by flow cytometry. Data represent the cell death fraction, normalizing the treatment with CLytA-DAAO as 1 ± SD with n ≥ 3. * indicates a *p*-value < 0.05, ** *p*-value < 0.01 and *** *p*-value < 0.001.

To identify potential regulatory mechanisms responsible for CLytA-DAAO-induced cell death, we studied some of the main signaling pathways affected by oxidative stress and DNA damage. Among the mitogen-activated protein kinase (MAPK) pathways, we have mainly focused on those leading to the activation of extracellular signal-regulated kinases (ERKs), c-Jun N-terminal kinases (JNK) and p38 mitogen-activated protein kinases (p38) [25]. We investigated whether activation or inhibition of these three kinases appears after short CLytA-DAAO and D-Ala treatments in a sensitive and a resistant cell line to CLytA-DAAO-induced cell death. In the sensitive cell line, IMIM-PC-2, a significant increase in phosphorylation of the three kinases took place as shown in Figure 4A. In contrast, the resistant cell line, Hs766T, only showed significant changes in JNK and p38. Strikingly, after 30 min of treatment, JNK phosphorylation increased while p38 phosphorylation decreased.

Next, we asked whether ERK, JNK and p38 kinases play a role in CLytA-DAAO-induced cell death. To this end, we pretreated cells with inhibitors of these three kinases in our cell models. We pretreated the cell lines with an inhibitor of the principal ERK activator, MEK1/2 (AZD6244), and a JNK inhibitor (SP600125). We were unable to detect a significant change in cell death induced by CLytA-DAO in any of the tested cell lines (Hs766T, IMIM-PC-2, RWP-1, HT-29, SW-480, HGUE-GB-37 and HGUE-GB-39) (data not shown). However, pretreating the cells with PD 169316, a P38 inhibitor, led to a general increase in CLytA-DAAO-induced cell death in all cell lines tested (Figure 4B). These results suggest that in response to CLytA-DAAO, cells display a stress and inflammatory defensive response, mediated by p38.

### 2.4. Molecular Mechanisms Involved in the Acquisition of Resistance to CLytA-DAAO-Induced Cell Death

CLytA-DAAO-induced cell death has been studied on several pancreatic and colorectal carcinoma and glioblastoma cell lines. The Hs766T pancreatic carcinoma cell line and HT-29 colorectal carcinoma cell line have been previously identified as resistant to CLytA-DAAO-induced cell death [11]. Furthermore, the resistance mechanism to induced cell death seems to be different between cell lines since the increase in intracellular reactive oxygen species (ROS) and DNA damage was greater in the Hs766T cell line as compared with the HT-29 cell line [11]. Hs766T presented a ROS increase of 2.25 ± 0.08-fold whereas HT-29 showed only 1.36 ± 0.03-fold after 2 h of treatment. On the other hand, histone H2A.X phosphorylation increased to 51.48 ± 2.93% in Hs766T and to 37.14 ± 3.67% in HT-29 with respect to the control after 3 h of treatment, indicating greater DNA damage in Hs766T than in HT-29 (data not shown). Therefore, we hypothesized that the resistant mechanism in HT-29 was mostly related to ROS detoxification, while in the Hs766T cell line the resistant mechanism was related to the reparation of cell damage induced by ROS.

**Figure 5:**
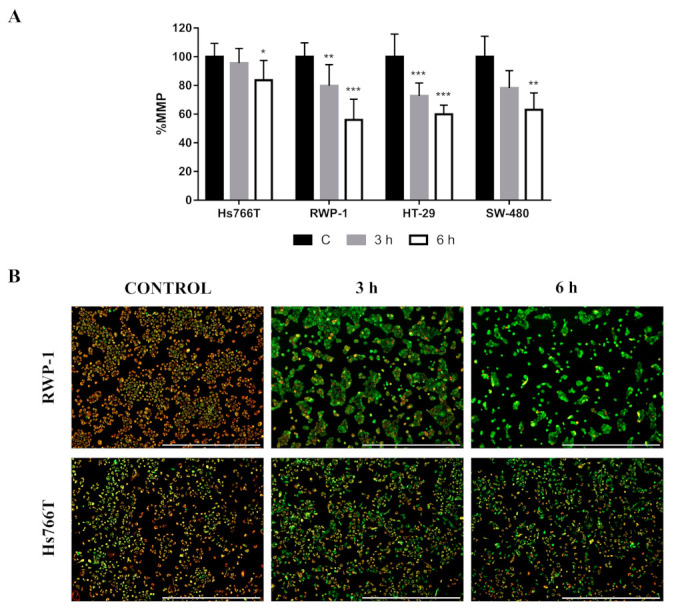
Role of MMP in resistance to CLytA-DAAO-induced cell death. (**A**) Hs766T, RWP-1, HT-29 and SW-480 cell lines were treated with CLytA-DAAO and D-Ala for 3 or 6 h and the MMP was determined using MitoTracker Green FM (all mitochondria staining) and MitoTracker Red CMXRos (polarized mitochondria staining) fluorescent dyes. Data represent the percentage of MMP, normalizing the control cells (C) as 100% ± SD with n ≥ 6. * indicates a *p*-value < 0.05, ** *p*-value < 0.01 and *** *p*-value < 0.001. (**B**) Representative fluorescent images of RWP-1 and Hs766T cell lines treated with CLytA-DAAO and D-Ala for 0, 3 or 6 h are shown. Scale bars, 1000 μm.

As shown above, a key phenomenon for CLytA-DAAO-induced cell death is mitochondrial membrane depolarization (Figure 1A). Therefore, we asked whether the MMP changes are correlated with cell line resistance to cell death. We have found that the MMP changes in HT-29 were quite similar to those observed in the sensitive cell lines RWP-1 and SW-480. However, in the Hs766T cell line the mitochondrial membrane depolarization was significantly lower than in CLytA-DAAO-induced cell death-sensitive cell lines (Figure 5A,B), suggesting that there is a direct relationship between cell line resistance and MMP changes.

Together, these findings suggest that there are a series of events taking place during CLytA-DAAO-induced cell death where an intracellular ROS increase is the first step. Then, the mitochondrial membrane is depolarized by calcium uptake by the mitochondria or another alternative mechanism. This depolarization leads to the release of AIF, cytochrome c and, probably, Endo-G by the mitochondria. Finally, DNA damage occurs as a consequence not only of the chromatin fragmentation caused by AIF (and Endo-G), but also by the direct effect of ROS on DNA. Therefore, we concluded that the Hs766T resistance mechanism to CLytA-DAAO-induced cell death is dual, inhibiting the MMP decrease and increasing DNA repair mechanisms.

**Figure 6:**
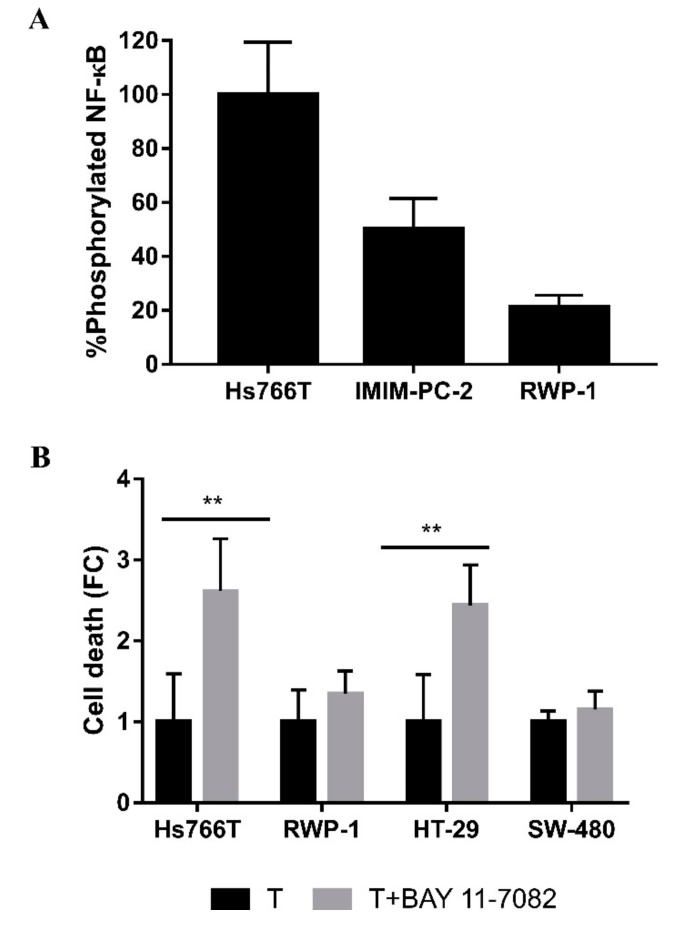
Role of NF-κB in the resistance to CLytA-DAAO-induced cell death. (**A**) Hs766T, IMIM-PC-2 and RWP-1 cell lines were treated with CLytA-DAAO and D-Ala for 1 h and NF-κB phosphorylation was determined by ELISA assay. Data represent the percentage of NF-κB phosphorylation, normalized with respect to Hs766T ± SD with n ≥ 3. (**B**) Hs766T, RWP-1, HT-29 and SW-480 cell lines were treated for 24 h with CLytA-DAAO and D-Ala (T) in the presence or absence of BAY 11-7082, an NF-κB inhibitor, and cell death was determined by flow cytometry. Data represent the cell death increase after NF-κB inhibition, normalizing the treatment with CLytA-DAAO as 1 ± SD with n ≥ 3. ** indicates a *p*-value < 0.01.

P38 is a MAPK activated in response to stress that initiates an inflammatory response [26,27]. Having demonstrated that the P38 inhibition significantly enhances the cell death in Hs766T (Figure 4), we next assessed the influence of the proinflammatory signaling pathway. We hypothesized that nuclear factor kappa B (NF-κB), a transcription factor closely related to cell survival and the inflammatory response [28], could have a pivotal role in the Hs766T resistance to CLytA-DAAO-induced cell death. NF-κB phosphorylation was determined in Hs766T and in two additional pancreatic carcinoma cell lines sensitive to CLytA-DAAO-induced cell death (IMIM-PC-2 and RWP-1) after being treated for 1 h. We found that the increase in NF-κB phosphorylation in the Hs766T cell line was higher than the one observed in the sensitive cell lines (Figure 6A). In addition, we used fluorescence microscopy to confirm NF-κB translocation to the nuclei after CLytA-DAAO treatment (Figure S2). We next examined the effect of NF-κB inhibition by pretreating pancreatic and colorectal carcinoma cell lines with BAY 11-7082 before CLytA-DAAO treatment. Interestingly, we found cell death significantly enhanced in both resistant cell lines, Hs766T and HT-29, while CLytA-DAAO-induced cell death in sensitive cell lines was not affected (Figure 6B).

**Figure 7:**
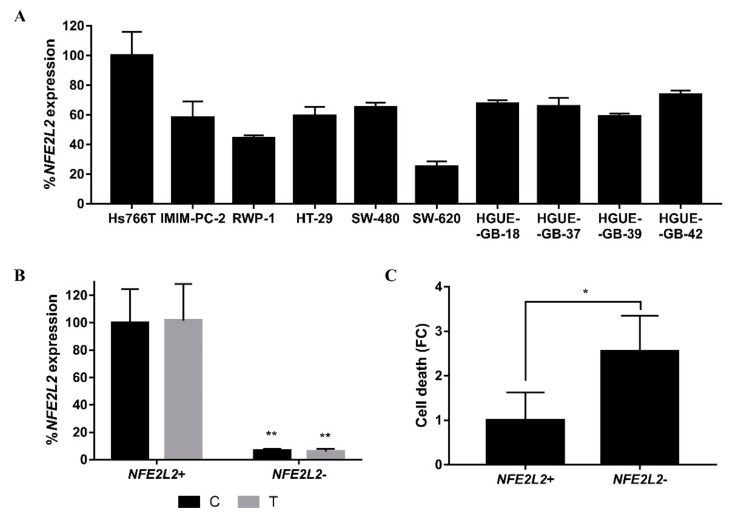
Role of *NFE2L2* in the resistance to CLytA-DAAO-induced cell death. (**A**) *NFE2L2* expression in all pancreatic and colon carcinoma and glioblastoma cell lines previously studied. Data represent the percentage of *NFE2L2* expression, normalized with respect to Hs766T ± SD with n ≥ 3. (**B**) *NFE2L2* expression levels in Hs766T cell line, control (C) and treated (T) with CLytA-DAAO and D-Ala, after being transfected with a non-specific (*NFE2L2*+) or an *NFE2L2* siRNA (*NFE2L2*−). Data represent the percentage of *NFE2L2* expression, normalized with respect to control transfected with a non-specific siRNA ± SD with n ≥ 3. (**C**) Fold change (FC) of Hs766T cell death after being treated with CLytA-DAAO and D-Ala for 24 h and transfected with a non-specific (*NFE2L2*+) or an *NFE2L2* siRNA (*NFE2L2*−). Data represent the cell death increase ± SD with n ≥ 3. * indicates a *p*-value < 0.05 and ** *p*-value < 0.01.

Nuclear factor erythroid 2-related factor 2 (Nrf2), encoded by the *NFE2L2* gene, is another transcription factor that has been related to the oxidative stress response, inflammation and survival [29]. We subsequently investigated *NFE2L2* expression in all cell lines previously studied. We found that Hs766T shows a higher expression as compared with the rest of the cell lines (Figure 7A). In order to understand the importance of *NFE2L2* in cell death, we specifically silenced the *NFE2L2* gene performing siRNA transfection. Overall, a successful blockade of *NFE2L2* expression in the Hs766T cell line (Figure 7B) led to an increase in CLytA-DAAO-induced cell death (Figure 7C).

**Figure 8:**
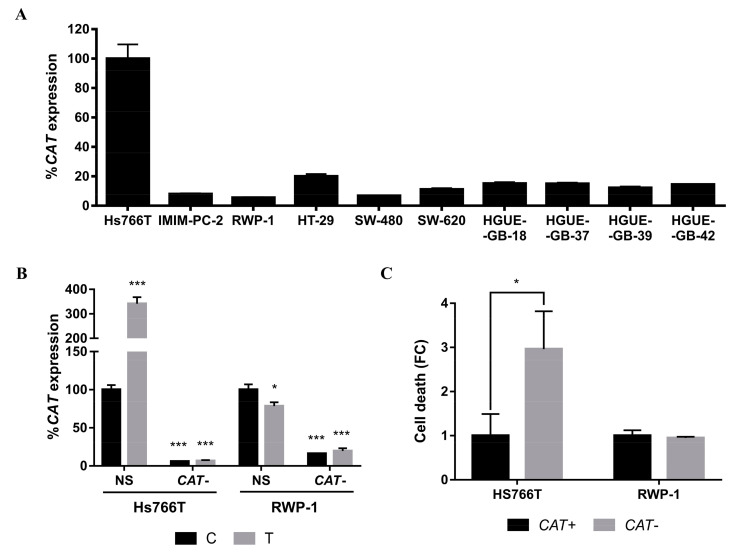
Role of *CAT* in the resistance to CLytA-DAAO-induced cell death. (**A**) *CAT* expression in pancreatic and colon carcinoma and glioblastoma cell lines. Data represent the percentage of catalase expression, normalized with respect to Hs766T ± SD with n ≥ 3. (**B**) *CAT* expression in Hs766T and RWP-1 cell lines, control (C) and treated with CLytA-DAAO and D-Ala (T), after being transfected with a non-specific (NS) or a catalase siRNA (CAT−). Data represent the percentage of catalase expression, normalized with respect to control transfected with a non-specific siRNA ± SD with n ≥ 3. (**C**) Hs766T and RWP-1 cell death after being treated with CLytA-DAAO and D-Ala and transfected with a non-specific (CAT+) or a *CAT* siRNA (CAT−) for 24 h. Data represent the cell death increase ± SD with n ≥ 3. * indicates a *p*-value < 0.05 and *** *p*-value < 0.001.

Detoxification enzymes are another factor to consider in the resistance to cell death. Given that cell death is generated by a protein that creates oxidative stress, we investigated the role of catalase, which is closely related to free radical detoxification [30]. Consistent with *NFE2L2* observations, catalase expression was higher in Hs766T than in the rest of the cell lines tested (Figure 8A). For this reason, catalase (*CAT*) expression was inhibited in Hs766T and RWP-1, a sensitive cell line to CLytA-DAAO-induced cell death. Inhibition was performed using siRNA and q-PCR assays to verify the results. Experiments with siRNA not only significantly blocked *CAT* expression in both cell lines, but they also showed that *CAT* expression increases three-fold in Hs766T while in RWP-1 decreases approximately by 20% after CLytA-DAAO treatment. Collectively, these results reinforce the important role of *CAT* in the resistance observed in Hs766T (Figure 8B). In order to verify whether changes in the effect induced by the CLytA-DAAO treatment were produced, we inhibited *CAT* expression and then we performed viability assays. After transfection, cell death was increased significantly in Hs766T treated with CLytA-DAAO, but not in the RWP-1 cell line (Figure 8C). In contrast to our initial hypothesis about a resistance mechanism based on detoxification in HT-29, no significant results with catalase were observed in this cell line (data not shown).

**Figure 9:**
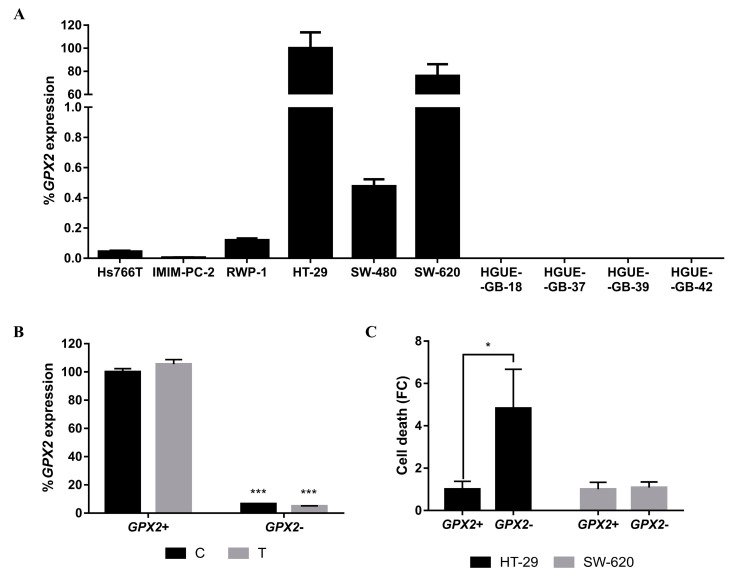
Role of GPX2 in the resistance to CLytA-DAAO-induced cell death. (**A**) *GPX2* expression in all cancer cell lines previously studied. Data represent the percentage of *GPX2* expression, normalized with respect to HT-29 ± SD with n ≥ 3. (**B**) *GPX2* expression levels in HT-29 cell line control (**C**) and treated with CLytA-DAAO and D-Ala (T), after being transfected with non-specific (*GPX2*+) or *GPX2* siRNA (*GPX2*−). Data represent the percentage of *GPX2* expression, normalized with respect to control transfected with non-specific siRNA ± SD with n ≥ 3. (**C**) HT-29 and SW-620 cell death after being treated with CLytA-DAAO and D-Ala for 24 h and transfected with non-specific (*GPX2*+) or *GPX2* siRNA (*GPX2*−). Data represent the percentage of cell death ± SD with n ≥ 3. * indicates a *p*-value < 0.05 and *** *p*-value < 0.001.

The role of glutathione peroxidase 2 (GPX2), an antioxidant enzyme, has been previously demonstrated in the proliferation and differentiation of the HT-29 cell line [31,32]. To analyze whether GPX2 has a role in resistance to CLytA-DAAO-induced cell death, *GPX2* expression was studied in all cell lines. Results show that this enzyme is highly expressed in HT-29 and other colorectal carcinoma cell lines while its expression is very low in pancreatic carcinoma and glioblastoma cell lines (Figure 9A). Although SW-620 is not resistant to CLytA-DAAO-induced cell death, its basal expression was also very high. Therefore, we performed a *GPX2* siRNA transfection assay in both cell lines to verify whether GPX2 is important in the resistance to CLytA-DAAO-induced cell death. Both cell lines transfected with a *GPX2* siRNA or a non-specific siRNA (Figure 9B) were treated with CLytA-DAAO and D-Ala. Cell death did not change in SW-620 whereas there was a significant cell death enhancement in HT-29 (Figure 9C), suggesting a possible participation of GPX2 in the resistance mechanism of HT-29.

**Figure 10:**
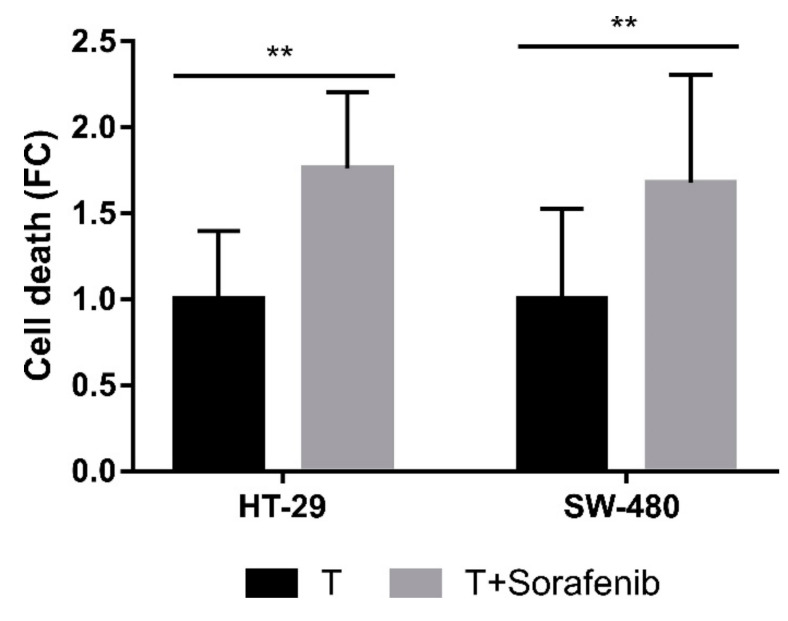
BRAF role in HT-29 resistance to CLytA-DAAO-induced cell death. HT-29 cell line was treated with CLytA-DAAO and D-Ala (T) in the presence or absence of sorafenib for 24 h and cell death was determined by flow cytometry. Data represent the cell death increase ± SD with n ≥ 3. ** indicates a *p*-value < 0.01.

BRAF, a member of the RAF family of serine/threonine kinases, is essential to the regulation of cellular growth, proliferation and survival. Mutations in the BRAF gene have been described in several types of tumors [33] and it is widely known that the HT-29 cell line has a mutated BRAF (V600E) while the other colorectal carcinoma cell lines tested (SW-480 and SW-620) harbor wild type BRAF [34,35,36]. In order to find out whether BRAF inhibition has an effect on the colorectal carcinoma resistant cell line HT-29, we used sorafenib as BRAF inhibitor. Probably due to the non-specific nature of the BRAF inhibitor, pretreatment with sorafenib resulted in an increased cell death in both HT-29 and SW-480 cell lines regardless of their BRAF status (Figure 10). Sorafenib can prevent the phosphorylation of other proteins, such as P38 [37], whose inhibition enhances the CLytA-DAAO effect, as described above. Therefore, in addition to detoxification, the inflammatory response may also be involved in HT-29 resistance.

## 3. Discussion

Recently, we have demonstrated the ability of CLytA-DAAO to induce cell death in pancreatic and colorectal carcinoma cell lines as well as in glioblastoma cell lines [11]. The CLytA-DAAO-induced cell death is associated with intracellular ROS increase and DNA damage analyzed through histone H2A.X phosphorylation. Indeed, we observed that glioblastoma cell lines were protected against CLytA-DAAO-induced death after pretreatment with a general caspase inhibitor, suggesting a classical apoptotic cell death. Regarding pancreatic and colorectal carcinoma cell lines, none of the traditional cell death inhibitors protect them from CLytA-DAAO-induced cell death, discarding apoptosis, autophagy, necroptosis and ferroptosis [11].

In this study we evaluated the role of the MMP in the cell death induction caused by CLytA-DAAO treatment. Results shown in Figure 1A indicate that mitochondrial membrane depolarization occurs during the first hours of treatment in all cell lines of the three cancer models. MMP decrease is produced by a sudden increase in the mitochondrial membrane permeability caused by the opening of the mitochondrial permeability transition pore (PTP) [38], suggesting that the cell death evoked by CLytA-DAAO in colon and pancreatic carcinoma is some type of MPT-driven necrosis [3]. It has been widely described that one of the main inducers of PTP opening is calcium [39,40,41]. Apoptosis-inducing factors usually increase levels of calcium in the cytosol, prior to the MMP decrease and the caspase activation. In fact, intracellular calcium chelators can inhibit apoptotic cell death [42,43]. Additionally, MPT-driven necrosis has been associated with cytoplasmic calcium overload [3].

To determine whether intracellular calcium mobilization plays a role in CLytA-DAAO-induced death, we preloaded the cells with BAPTA/AM, a calcium chelator, to inhibit any intracellular calcium mobilization when CLytA-DAAO was added. To discard any effect mediated by extracellular calcium uptake, we performed parallel experiments where EGTA was added to the cell culture medium to eliminate calcium before CLytA-DAAO addition. The results showed that only BAPTA/AM had a protective effect (Figure 1B), demonstrating the importance of intracellular calcium movement within the cell to trigger the CLytA-DAAO-induced cell death.

Intracellular calcium is mainly stored in endoplasmic reticulum (ER). Recent studies reported that an uncontrolled ROS increase, such as that produced by the reaction catalyzed by CLytA-DAAO, causes ER stress resulting in calcium release through the inositol-1,4,5-triphosphate receptor (IP3R) and its entrance to the mitochondria [13,44,45]. In future studies, the relationship between CLytA-DAAO-induced cell death and ER stress should be addressed.

**Figure 11:**
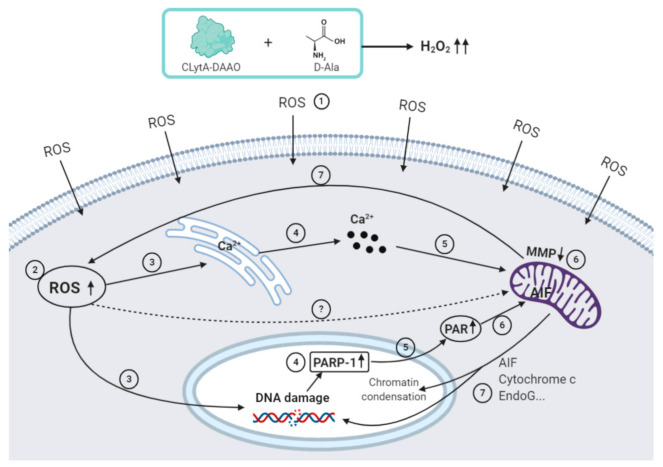
The general mechanism of cell death induced by CLytA-DAAO. The reaction catalyzed by CLytA-DAAO when oxidizing D-Ala generates H_2_O_2_, a potential reactive oxygen species (ROS) producer. ROS enters the cell (1) and its accumulation inside the cell (2) induces a calcium release from the endoplasmic reticulum (ER) (3) to the cytosol (4) through IP3R channels and direct damage to DNA (3). The calcium released is directed to the mitochondria (5), causing mitochondrial membrane depolarization (6) and, consequently, promoting additional ROS production (7). In turn, the MMP decrease is associated with the exit of proteins from the mitochondria, which finally causes DNA fragmentation and chromatin condensation, such as AIF, cytochrome c, Endo G, etc. (7). On the other hand, when the ROS-induced DNA damage is very high, there is an overactivation of PARP-1 (4), which leads to an accumulation of the PAR polymer in the cytosol (5), which also causes AIF translocation from the mitochondria to the nucleus (6). The results obtained in IMIM-PC-2 suggest that, in addition, ROS are capable of acting directly on the mitochondrial membrane by an unknown mechanism (?), since depolarization is produced but calcium does not play a role in cell death (Created with BioRender.com).

Altogether, our results reveal different cell death mechanisms triggered by CLytA-DAAO and D-Ala treatment depending on the cellular context; so far, apoptotic cell death in glioblastoma and some type of MPT-driven necrosis in colon and pancreatic carcinomas. Broadly, we found that the mitochondrial membrane depolarization is crucial in all cell models tested. Moreover, our results suggest that this decrease in MMP is mediated by the entry of calcium into the mitochondria in most cases. The different cell death mechanisms observed are explained in detail below and a general scheme of the cell death mechanism induced by CLytA-DAAO is displayed in Figure 11.

Regarding glioblastoma cell lines, in a previous work we already stated that a pan-caspase inhibitor is capable of protecting against CLytA-DAAO-induced cell death and Bax inhibition generates partial protection [11]. The two main types of apoptosis are those induced by extrinsic and intrinsic pathways. However, mitochondria play a more important role in the intrinsic pathway, which is mediated by DNA damage, calcium overload and oxidative stress. In this pathway, cytochrome c is released from the cytosol and assembles with Apaf-1, procaspase-9 and dATP, causing caspase-9 activation [46]. Furthermore, in the intrinsic pathway, proapoptotic proteins such as AIF, EndoG, Smac, Omi, etc. are released from the mitochondria and AIF and EndoG are translocated to the nucleus, where they induce chromatin condensation and DNA fragmentation [47].

We have observed that mitochondrial membrane depolarization occurs in glioblastoma (Figure 1A) and CLytA-DAAO-induced cell death is partially dependent on intracellular calcium movement in HGUE-GB-37. However, the partial protection due to its inhibition with BAPTA/AM does not occur in HGUE-GB-39 (Figure 1B). Next, analysis of the AIF location by immunocytochemistry shows that AIF translocation to the nucleus is produced in both cell lines (Figure 3). Therefore, our results suggest that the intrinsic pathway plays a key role. Furthermore, a caspase-8 inhibitor has only a very modest effect on CLytA-DAAO-induced cell death in glioblastoma cell lines (data not shown), confirming that the intrinsic apoptosis pathway is the principal mechanism involved in CLytA-DAAO-induced cell death in glioblastoma.

Regarding the colon and pancreatic carcinoma cell lines, to try to more precisely define the cell death mechanisms evoked by CLytA-DAAO, we determined the effect of PARP-1 inhibition. It is well known that PARP-1 plays a dual role in DNA damage response (DDR). On one hand, when damage is slight, PARP-1 activation participates in the DNA base-excision repair system. On the other hand, when intense damage occurs, PARP-1 overactivation induces a cytotoxic PAR polymer accumulation in the cytosol leading to cell death [14]. We recently published work showing that, after the inhibition of PARP-1, an enhancement of the CLytA-DAAO effect can be observed in glioblastoma cell lines [11]. This implies that PARP-1 is essentially participating in DNA damage repair in glioblastoma cell lines. This prompted us to study the role of PARP-1 in CLytA-DAAO-induced cell death in pancreatic and colorectal carcinoma cell lines. In order to answer this question, we used DPQ, a PARP-1 inhibitor, in combination with CLytA-DAAO treatment. CLytA-DAAO-induced cell death was significantly blocked when PARP-1 was inhibited in RWP-1, SW-480 and SW-620 cell lines but not in IMIM-PC-2 (Figure 2).

Our results reveal different cell death mechanisms triggered by CLytA-DAAO and D-Ala treatment in pancreatic and colorectal carcinoma cell lines. In RWP-1, we observed a protective effect of DPQ (Figure 2), AIF translocation from mitochondria to the nucleus (Figure 3), the requirement of intracellular calcium mobilization for induced cell death (Figure 1B), and the consequent mitochondrial membrane depolarization (Figure 1A). Together, the present findings support the idea that in the RWP-1 cell line the MPT-driven necrosis could be more precisely described as a necrosis-like cell death induced by oxidative stress and DNA damage called parthanatos.

The calcium influx into the mitochondria causes mitochondrial membrane depolarization triggering a greater release of ROS from the mitochondria [48]. This process contributes to the final parthanatos cell death [13]. On the other hand, SW-480 and SW-620 seem to undergo another non-apoptotic cell death, which has also been reported, induced by oxidative stress, PARP-1 overactivation, calcium mobilization and it is AIF independent [12].

Finally, DPQ pretreatment did not induce significant changes in CLytA-DAAO-induced cell death in IMIM-PC-2 (Figure 2). Indeed, AIF did not enter the nucleus but kept surrounding it (Figure 3). For this cell line, we propose a caspase-independent programmed cell death that has been described in which AIF is released from mitochondria and is accumulated in the perinuclear region colocalized with chromatin at early stages [22,23,24].

To further characterize CLytA-DAAO-induced cell death mechanisms, we studied the role of different signal transduction pathways in cell death induction as well as in the resistance observed in some cellular models.

We have studied whether the MAPK signal pathways plays a role in the CLytA-DAAO-induced cell death. This pathway is involved in the regulation of many biological processes and it is usually altered in pathological conditions such as cancer [49]. It is known that oxidative stress and DNA damage activate the DDR, which is normally regulated by MAPKs [25]. Mammalian MAPKs include ERKs, JNKs and p38. ERKs are the mitogenic response pathway, mainly related to cell proliferation, survival and differentiation [50]. Instead, JNK and p38 are activated in response to stress and participate in several biological processes—survival, cell death and inflammatory processes, among others [51,52].

In the IMIM-PC-2 cell line, which is sensitive to CLytA-DAAO-induced cell death, the activation of the three MAPKs was observed, being more pronounced in JNK and P38 phosphorylation than that of ERK. Instead, in the Hs766T cell line, which is resistant to the effects of CLytA-DAAO, changes in activation levels were quite modest. Even more, JNK activation increased while p38 activation decreased and no changes in ERK phosphorylation levels were observed (Figure 4A). In order to evaluate the impact of these changes in CLytA-DAAO-induced death in all three cancer models, inhibitors against ERK, JNK and p38 were tested with CLytA-DAAO and D-Ala treatment. Neither pretreatment with an MEK1/2 inhibitor (AZD6244) nor with a JNK inhibitor (SP600125) produced significant variations in CLytA-DAAO-induced cell death in any of the cell lines tested (data not shown). This suggests that the JNK overactivation observed in IMIM-PC-2 is probably an initial survival attempt to repair the damage caused by the treatment. However, we found that p38 plays a crucial protective role against CLytA-DAAO treatment in both sensitive and resistant cell lines (Figure 4B). This finding is in accordance with previous reports linking the p38 signaling pathway with poor prognosis and with resistance to tamoxifen, androgen deprivation therapy, trastuzumab, cisplatin, etc. [53].

We next sought to gain insight into the CLytA-DAAO-induced cell death by studying the resistance mechanisms observed in some cell lines. We have recently published that some cell lines are totally or partially resistant to CLytA-DAAO-induced cell death. Among all the studied cell lines, Hs766T, a pancreatic carcinoma cell line, and HT-29, a colorectal carcinoma cell line, were the most resistant [11]. We established that there is an increase in ROS levels and histone H2A.X phosphorylation, related to DNA damage, after CLytA-DAAO treatment in Hs766T, but not in HT-29 [11]. This suggests a different resistance mechanism in both cell lines—one focused on the damage response and repair, and the other associated with ROS detoxification.

Furthermore, in the current study we have shown that intracellular calcium mobilization (Figure 1B) and mitochondrial membrane depolarization (Figure 1A) are also necessary for CLytA-DAAO-induced death. Nevertheless, Hs766T was the only resistant cell line that shows a lower MMP decrease in comparison with sensitive cell lines (Figure 5), suggesting that its resistance mechanism is related to the damage response and repair. Interestingly, previous studies have proved that the Hs766T cell line is resistant to common treatments such as gemcitabine, 5-fluoracil or cisplatin [54]. Additionally, unlike the rest of the tumor cell lines tested, this cell line does not present a p53 mutation [55,56], which gives it a greater response to drugs that induce DNA damage [57,58].

Taking into consideration that p38 inhibition significantly enhances death in Hs766T (Figure 4B) and p38 is related to the oxidative stress response and inflammation, we next analyzed NF-κB’s role in resistance. NF-κB is a transcription factor also related to survival and inflammation which is additionally regulated by p38 phosphorylation [59,60]. After CLytA-DAAO treatment, Hs766T showed a higher NF-κB activation as compared to CLytA-DAAO-sensitive cell lines (Figure 6A). Consistently, NF-κB inhibition resulted in a significant enhancement of Hs766T cell death (Figure 6B).

Another signaling pathway activated in the stress response is Nrf2. Under normal conditions, Nrf2 is bound to Keap1, and when cells are exposed to stress, the dimer is split and Nrf2 targets the nucleus to activate the expression of genes related to the antioxidant response [61]. It is described that the separation of Nrf2 and Keap1 occurs through the phosphorylation of Nrf2, caused by ERK and p38 [62]. *NFE2L2* is the gene that encodes Nrf2 and it is basally more expressed in Hs766T than in the rest of pancreatic and colorectal carcinoma and glioblastoma cell lines (Figure 7A). Furthermore, the transfection of siRNA molecules against *NFE2L2* followed by CLytA-DAAO treatment (Figure 7B) increases sensitivity to cell death (Figure 7C). Although it is described that there is an inverse relationship between Nrf2 and NF-κB [63], our results reveal that the high expression and/or activation of both are necessary to protect cells from the CLytA-DAAO effect.

Expanding our analyses to the detoxification mechanism, we studied catalase as the main antioxidant enzyme. Interestingly, basal catalase overexpression was observed in Hs766T but not in HT-29 (Figure 8A). Moreover, after treatment with CLytA-DAAO, catalase levels increased about three-fold (Figure 8B) and blocking catalase expression with a specific siRNA led to a significant increase in CLytA-DAAO-induced death (Figure 8C). Consistently with catalase expression, siRNA transfection against catalase in HT-29 resulted in no significant changes in CLytA-DAAO-induced death (data not shown).

Overall, our results suggest that in Hs766T the calcium entry into the mitochondria and the consequent MMP decrease does not occur and its resistance is mediated by a high expression and/or activation of p38, NF-κB, Nrf2 and catalase. All these factors are related to ER stress, which leads us to presume that Hs766T has a high basal ER stress, which causes its resistance to the oxidative stress generated by CLytA-DAAO treatment. The relationship among catalase, Nrf2, NF-κB, p38 and ER stress is shown in Figure S3. Other authors have described that high ER stress may be related to resistance to different drugs used in the treatment of breast cancer [64], chronic myelogenous leukemia [65] and cutaneous melanoma [66]. The ER stress response is mediated by three proteins located in the ER membrane: inositol-requiring 1 alpha (IRE1α), double-strand RNA-activated protein kinase-like ER kinase (PERK) and activating transcription factor 6 (ATF6) [67]. However, most articles relate IRE1α with the ER stress-mediated drug resistance [65,66]. IRE1α is responsible for the splicing of x-box binding protein 1 (XBP1) [68]. Therefore, we measured the expression levels of the transcription factor XBP1 that were overexpressed in Hs766T in comparison to the rest of cell lines (data not shown). Further analysis will be needed to confirm whether Hs766T resistance is mediated by increased basal ER stress with respect to the rest of sensitive cell lines.

The role of GPX2, a detoxification enzyme, has been previously described in HT-29 cell survival [31,32]. Notably, a V600E mutation in BRAF, a serine/threonine-protein kinase that is involved in the RAS-RAF-MEK-ERK signaling pathway, is considered a poor prognosis marker that induces resistance to many therapies [36,69,70]. When V600E mutations exist in BRAF, BRAF is constitutively activated, causing the continued phosphorylation of MEK and ERK and thus, promoting the growth, proliferation, differentiation, migration and survival of tumor cells [70].

Moreover, we observed that *GPX2* was highly expressed in HT-29 and SW-620 (Figure 9A) and *GPX2* expression inhibition with a specific siRNA (Figure 9B) sensitizes HT-29 to the CLytA-DAAO effect (Figure 9C). On the other hand, pretreating cells with sorafenib results in an increase in the CLytA-DAAO effect, but there was no difference between the potentiation observed in HT-29 and SW-480 (Figure 10). These findings suggest that BRAF mutation is not responsible for its resistance. This conclusion is in accordance with the result obtained with the AZD6244 pretreatment, in which no modifications in cell death were obtained after inhibiting MEK (data not shown). We have shown an increase in cell death in HT-29 through inhibition of both P38 (Figure 4B) and NF-κB (Figure 6B), which suggests the participation of the inflammatory response in resisting CLytA-DAAO-induced cell death. It has been demonstrated that sorafenib is a poorly selective BRAF inhibitor and that one of the targets is p38 [37]. Thus, HT-29 resistance is mediated both by GPX2, an antioxidant protein, and by p38 and NF-κB, which participate in the inflammatory response against oxidative damage.

This work highlights the fact that CLytA-DAAO induces apoptotic and necrosis-like cell death depending on the cellular context. We have identified several cell death-induced mechanisms that take place on the tumor cell lines studied. This capacity to adapt to the environment of each cancer type and induce cell death by different mechanisms give CLytA-DAAO treatment a broad spectrum of putative cell targets, increasing the possibility to use the enzymes in many types of cancer cells. Our previous observation, that there is almost no effect on non-tumoral cell models such as fibroblasts, pancreatic cells, lymphocytes and adipocytes, further supports this idea. Similarly, resistance to CLytA-DAAO-induced death is not due to the presence of a single mechanism capable of blocking the damage generated, but rather to the interconnection of several pathways.

Finally, these data, while providing a unique tool against tumor cells, also need to be tested in patients. Therefore, we are currently starting a pilot study using biopsies from patients with pancreatic or colorectal carcinomas and glioblastomas. We will analyze the expression of the identified genes to evaluate the expression profile of the identified genes involved in the resistance to CLytA-DAAO-induced cell death in cancer patients. Knowledge of the mechanisms and pathways involved in both CLytA-DAAO-induced death and resistance is pivotal for this enzyme treatment to ultimately become an effective anticancer therapy.

## 4. Materials and Methods

### 4.1. Cell Culture

RWP-1, IMIM-PC-2 and Hs766T pancreatic adenocarcinoma cell lines, SW-480, SW-620 and HT-29 colorectal carcinoma cell lines and HGUE-GB-18, HGUE-GB-37, HGUE-GB-39 and HGUE-GB-42 glioblastoma cell lines were used. Glioblastoma cell lines were derived from primary cell cultures established by our group in the “Hospital General Universitario de Elche” (HGUE) [71]. All cell lines used in this study were maintained as previously described [11].

### 4.2. Chemical Reagents

In total, 2 units mL^−1^ (U mL^−1^) of CLytA-DAAO in combination with 1 mM D-Alanine (Alfa Aesar, Thermo Fisher Scientific, Kandel, Germany) were used. CLytA-DAAO was obtained and isolated from *Escherichia coli* BL21 (DE3), transformed with the plasmid pCPC21. The isolation process of CLytA-DAAO and the measurement of its enzymatic activity have been previously described [11].

All the chemical reagents mentioned below were used to study the mechanism of cell death induced by CLytA-DAAO.

To analyze the role of calcium in CLytA-DAAO-induced cell death, 5 μM 1,2-Bis(2-aminophenoxy)ethane-N,N,N’,N’-tetraacetic acid tetrakis(acetoxymethyl ester) (BAPTA/AM) (Sigma-Aldrich, St. Louis, MO, USA), an intracellular calcium chelator, and 100 μM ethylene glycol-bis(2-aminoethylether)-N,N,N’,N’-tetraacetic acid (EGTA) (Sigma-Aldrich, St. Louis, MO, USA), an extracellular calcium chelator, were used.

To evaluate whether PARP-1 participates in CLytA-DAAO-induced cell death, 10 μM 3,4-Dihydro-5(4-(1-piperindinyl)butoxy)-1(2H)-isoquinoline (DPQ) (Calbiochem^®^, San Diego, CA, USA), a PARP inhibitor III, was tested.

In total, 10 μM 4-(4-Fluorophenyl)-2-(4-nitrophenyl)-5-(4-pyridyl)-1H-imidazole (PD 169316) (Calbiochem^®^, San Diego, CA, USA), a p38 MAP kinase inhibitor, was used to demonstrate the role of p38 in cell death induced by CLytA-DAAO.

Finally, 5 μM (E)-3-(4-Methylphenylsulfonyl)-2-propenenitrile (BAY 11-7082) (Sigma-Aldrich, St. Louis, MO, USA), an NF-κB inhibitor and 1 μM N-(4-Chloro-3-(trifluoromethyl)phenyl)-N’-(4-(2-[N-methylcarbamoyl]-4-pyridyloxy)phenyl)urea (sorafenib) (Sigma-Aldrich, St. Louis, MO, USA), were used to define the role of NF-κB and BRAF in the resistance to cell death observed in Hs766T.

The previous reagents, with the exception of BAPTA/AM, were added 30 min before treatment with CLytA-DAAO and D-Ala and kept in the medium throughout the treatment time. BAPTA/AM was added 1 h before CLytA-DAAO and was withdrawn from the medium after this time.

### 4.3. Cell Death

Cell death was determined through the permeability of the plasmatic membrane; for this purpose, six-well plates (Sarstedt, Nümbrecht, Germany) were used. After the treatment time, cells were harvested by trypsinization and the Muse^®^ Count & Viability Kit (Luminex^®^, Austin, TX, USA) was added following the manufacturer’s instructions. Cell death was determined using the Muse^®^ Cell Analyzer (Luminex^®^, Austin, TX, USA).

### 4.4. Mitochondrial Membrane Potential

MMP was evaluated using two fluorescent probes: MitoTracker Red CMXRos (Molecular Probes, Eugen, OR, USA), capable of labeling all mitochondria that conserve MMP, and MitoTracker Green CMXRos (Molecular Probes, Eugen, OR, USA), which labels mitochondria independently of the MMP.

Cells were seeded and treated in 96-well plates (Sarstedt, Nümbrecht, Germany). After the treatment time, cells were incubated for 30 min in media with 200 nM MitoTracker Red CMXRos and 200 nM de MitoTracker Green CMXRos. Finally, fluorescence was quantified using Cytation 3 Cell Imaging Multi-mode (BioTek, Winooski, VT, USA).

### 4.5. Immunocytochemistry

Cells were seeded on glass coverslips located in 24-well plates (Sarstedt, Nümbrecht, Germany), and treated or not with 2 U/mL CLytA-DAAO and 1 mM D-Ala for 6 h. Then, cells were washed with PBS 1X three times and fixed with 4% paraformaldehyde solution for 10 min at room temperature. Next, to permeate the cells, 0.2% Triton X-100 (Sigma Aldrich, St. Louis, MO, USA) solution was added for 10 min with gentle shaking and, subsequently, the non-specific binding sites were blocked by incubating cells in a 1% bovine serum albumin (BSA, Sigma Aldrich, St. Louis, MO, USA) solution for 1 h in agitation. After each incubation, three washes with PBS 1X were performed.

AIF protein was marked using anti-AIF (#4642, Cell Signaling Technology, Danvers, MA, USA). Cells were incubated with a blocking buffer (5% rabbit serum and 0.3% Triton X-100 in PBS 1X) containing the AIF antibody (1%) overnight. As a secondary antibody, a rabbit anti-IgG marked with Alexa Fluor 488 (Molecular Probes, Eugene, OR, USA) was added at 1% to blocking buffer and nuclei were marked with 4′,6-diamidino-2-phenylindole (DAPI). Cells were incubated for 1 h in darkness. After staining, cells were observed and photographed with a confocal microscope—Leica TCS SP2 (Leica Microsystems, Wetzlar, Germany).

### 4.6. ELISA Assay

The MAPK Family (ERK, p38, JNK) Activation InstantOne ELISA™ Kit (Invitrogen, Carlsbad, CA, USA) and phospho-NFkB p65 (Ser536) InstantOne ELISA™ Kit (Invitrogen, Carlsbad, CA, USA) were used. Cells were seeded in a T25 culture flask (Sarstedt, Nümbrecht, Germany) and, once the treatment was finished, the instructions provided by the manufacturer were followed. Finally, the absorbance at 450 nm was quantified on a Gen5™ microplate reader (BioTek, Winooski, VT, USA).

### 4.7. Gene Expression Analysis

RNA isolation was performed using an NZY Total RNA Isolation kit (NZYtech, Lisbon, Portugal) according to the manufacturer’s instructions. A total of 1 µg of the obtained RNA was transformed into cDNA using the High-Capacity cDNA Reverse Transcription Kit (Applied Biosystems, Foster City, CA, USA) following the manufacturer’s instructions.

Quantitative polymerase chain reaction (qPCR) was performed using 1 µl predesigned TaqMan^®^ Gene Expression Assays (Applied Biosystems, Foster City, CA, USA), 10 µL NZYSpeedy qPCR Probe Master Mix (2x), ROX plus (NZYtech, Lisbon, Portugal) and 4 µL cDNA, which was previously obtained. The Taqman Gene Expression Assays used to measure the expression of the following genes were *CAT* (Hs00156308_m1), *NFE2L2* (Hs00975961_g1), *GPX2* (Hs01591589_m1) and *GAPDH* (Hs02786624_g1) as endogenous controls. The 7300 Real Time PCR System (Applied Biosystems, Foster City, CA, USA) was used.

### 4.8. Small Interfering RNA (siRNA) Assay

To inhibit gene expression, cells were transfected with specific siRNA against *CAT*, *NFE2L2* and *GPX2*, and a non-specific siRNA (Invitrogen, Carlsbad, CA, USA) was used as a control. Cells were seeded into 6-well plates (Sarstedt, Nümbrecht, Germany) using media without antibiotic, and a solution of Opti-MEM™ I Reduced Serum Medium (Gibco, Life Technologies, Carlsbad, CA, USA) containing 2.5 µL/mL Lipofectamine™ RNAiMAX Transfection Reagent (Invitrogen, Carlsbad, CA, USA) and 5 pmol/mL siRNA was added.

The transfection was carried out for 48 h at 37 °C and then, the corresponding treatment was added. Once the treatment finished, cell death and gene expression were analyzed as described above.

### 4.9. Statistical Analysis

All results are represented as the mean ± standard deviation (SD) of at least three independent data. Statistical analysis was performed using the GraphPad Prism version 7 (GraphPad Software Inc., San Diego, CA, USA), as described in our previous work [11].

## 5. Conclusions

CLytA-DAAO-induced cell death is a phenomenon that is dependent on the cellular context. In glioblastoma cell lines a classical apoptosis mediated by the intrinsic pathway occurs. We have identified several types of necrotic-like cell death being induced in pancreatic and colorectal carcinoma cell lines. Independently of the cell death type induced by CLytA-DAAO, there are several key players that participate in cell death. In all cell lines there is an intracellular increase in ROS, DNA damage and mitochondrial membrane depolarization. On the other hand, intracellular calcium mobilization, PARP-1 and AIF also play a role in most of the cell models tested.

In addition, although the role of MAPKs pathways has been studied by ELISA and using specific inhibitors, only the p38 pathways appear to play a crucial role in CLytA-DAAO-induced cell death.

Finally, Hs766T resistance to CLytA-DAAO-induced cell death is mediated by antioxidant proteins, such as catalase and Nrf2, and proteins related to survival, stress and inflammatory response such as NF-κB and p38. HT-29 resistance is mediated not only by NF-κB and p38, but also by GPX2, an antioxidant protein.

## Supplementary Materials

Supplementary Materials can be found at https://www.mdpi.com/1422-0067/21/22/8522/s1, Figure S1: CLytA-DAAO-induced AIF translocation to the nucleus. AIF levels into the nucleus in RWP-1, IMIM-PC-2, SW-480, SW-620, HGUE-GB-37 and HGUE-GB-39 control (C) and treated (T) with 2 U/mL CLytA-DAAO and 1 mM D-Ala for 6 h. Green fluorescence in the nuclear area was quantified using ImageJ software. Data represent the fold-change (FC) values ± SEM of the green fluorescence intensity in the nucleus after normalized to the control cells. *** indicates a *p*-value < 0.001, Figure S2: CLytA-DAAO-induced NF-κB translocation to the nucleus. Images show RWP-1 and Hs766T cell lines control and treated with 2 U/mL CLytA-DAAO and 1 mM D-Ala for 1 and 6 h. Immunocytochemistry was performed by labeling NF-kB with anti-p65 (RelA) (Thermo Scientific) and nuclei were marked with HOESCHT. Images were taken with a fluorescence microscope (Nikon Eclipse TE2000-U) equipped with a digital camera (Nikon DS-1QM). NF-kB is displayed in red and nuclei in blue. Scale bars, 100 μM, Figure S3: Schematic representation showing the relationship between some of the genes involved in the Hs766T resistance to CLytA-DAAO-induced cell death and ER stress response. PERK, IRE1 and ATF6 are activated under ER stress conditions. ATF6 migrates to Golgi apparatus and suffers proteolytic activation by S1P and S2P proteases. Then, the processed ATF6 induces XBP-1 mRNA expression, which is processed by IRE-1 to render the active form, which is translocated to the nucleus, where it induces transcriptionally several genes, one of which is catalase. Additionally, IRE1 recruits TRAF2 to activate proteins related to the answer to stress and inflammation, such as P38, JNK and NF-κB. Finally, PERK phosphorylates NRF2 and EIF2α, which promotes the translation of ATF4. NRF2 and ATF4 are transcription factors that regulate genes that participate in the antioxidant response. Created with Biorender.com.

## Abbreviations

DAAO	D-amino acid oxidase
CLytA	Choline-binding module of the amidase N-acetylmuramoyl-L-alanine
DEAE	Diethylaminoethanol
PARP-1	Poly (ADP-ribose) polymerase 1
AIF	Apoptosis-inducing factor
MMP	Mitochondrial membrane potential
MAPK	Mitogen-activated protein kinase
ERK	Extracellular signal-regulated kinase
JNK	c-Jun N-terminal kinase
P38	P38 mitogen-activated protein kinase
ROS	Reactive oxygen species
NF-κB	Nuclear factor kappa B
Nrf2	Nuclear factor erythroid 2-related factor 2
CAT	Catalase
GPX2	Glutathione peroxidase 2
PTP	Permeability transition pore
ER	Endoplasmic reticulum
IP3R	Inositol-1,4,5-triphosphate receptor
DDR	DNA damage response
IRE1α	Inositol-requiring 1 alpha
PERK	Double-strand RNA-activated protein kinase-like ER kinase
ATF6	Activating transcription factor 6
XBP1	X-box binding protein 1
HGUE	*Hospital General Universitario de Elche* (General University Hospital of Elche)
BAPTA/AM	1,2-Bis(2-aminophenoxy)ethane-N,N,N’,N’-tetraacetic acid tetrakis(acetoxymethyl ester)
EGTA	Ethylene glycol-bis(2-aminoethylether)-N,N,N’,N’-tetraacetic acid
DPQ	3,4-Dihydro-5(4-(1-piperindinyl)butoxy)-1(2H)-isoquinoline
PD 169316	4-(4-Fluorophenyl)-2-(4-nitrophenyl)-5-(4-pyridyl)-1H-imidazole
BAY 11-7082	(E)-3-(4-Methylphenylsulfonyl)-2-propenenitrile
Sorafenib	N-(4-Chloro-3-(trifluoromethyl)phenyl)-N’-(4-(2-[N-methylcarbamoyl]-4-pyridyloxy)phenyl)urea
DAPI	4′,6-diamidino-2-phenylindole
qPCR	Quantitative polymerase chain reaction
SEM	Standard Error of the Mean
