# Supplementary material for: Cell Death Mechanisms Induced by CLytA-DAAO Chimeric Enzyme in Human Tumor Cell Lines
Source: Int J Mol Sci. 2020 Nov 12;21(22):8522. doi: 10.3390/ijms21228522 (PMC7697521; doi:10.3390/ijms21228522)
Supplement: Supplementary file 1 [file ijms-21-08522-s001.pdf]

Figure S1. CLytA-DAAO-induced AIF translocation to the nucleus. AIF levels into the nucleus in RWP-1, IMIM-PC-2, SW-480, SW-620, HGUE-GB-37 and HGUE-GB-39 control (C) and treated (T) with 2 U/mL CLytA-DAAO and 1 mM D-Ala for 6 h. Green fluorescence in the nuclear area was quantified using ImageJ software. Data represent the fold-change (FC) values ± SD of the green fluorescence intensity in the nucleus after normalized to the control cells. *** indicates a p-value < 0.001.

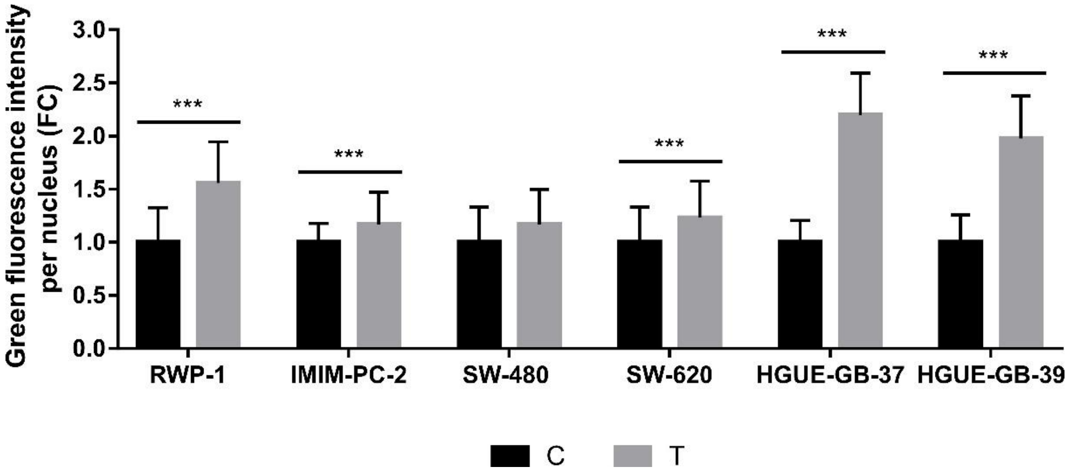

Figure S2. CLytA-DAAO-induced NF-κB translocation to the nucleus. Images show RWP-1 and Hs766T cell lines control and treated with 2 U/mL CLytA-DAAO and 1 mM D-Ala for 1 and 6 h. Immunocitochemistry was performed labeling NF-kB with anti-p65 (RelA) (Thermo Scientific) and nuclei were marked with HOESCHT. Images were taken with a fluorescence microscope (Nikon Eclipse TE2000-U) equipped with a digital camera (Nikon DS-1QM). NF-kB is displayed in red and nuclei in blue. Scale bars, 100 μM.

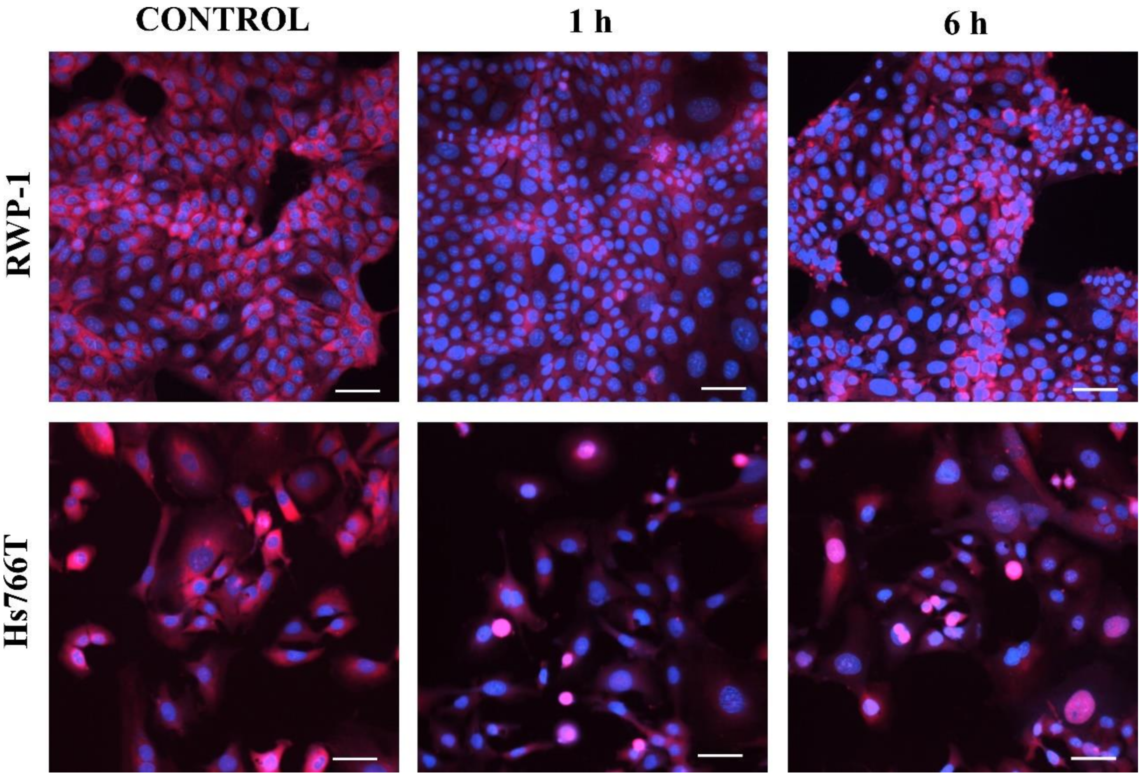

Figure S3. Schematic representation showing the relationship between some of the genes involved in the Hs766T resistance to CLytA-DAAO-induced cell death and ER stress response. PERK, IRE1 and ATF6 are activated under ER stress conditions. ATF6 migrates to Golgi apparatus and suffers proteolytic activation by S1P and S2P proteases. Then, the processed ATF6 induces XBP-1 mRNA expression, that is processed by IRE-1 to render the active form, which is translocated to the nucleus, where it induces transcriptionally several genes, one of which is catalase [1]. Additionally, IRE1 recruits TRAF2 to activate proteins related to the answer to stress and inflammation, such as P38, JNK and NF-κB [2]. Finally, PERK phosphorylates NRF2 and EIF2α, which promotes the translation of ATF4 [3,4]. NRF2 and ATF4 are transcription factors that regulate genes that participate in the antioxidant response. Created with Biorender.com.

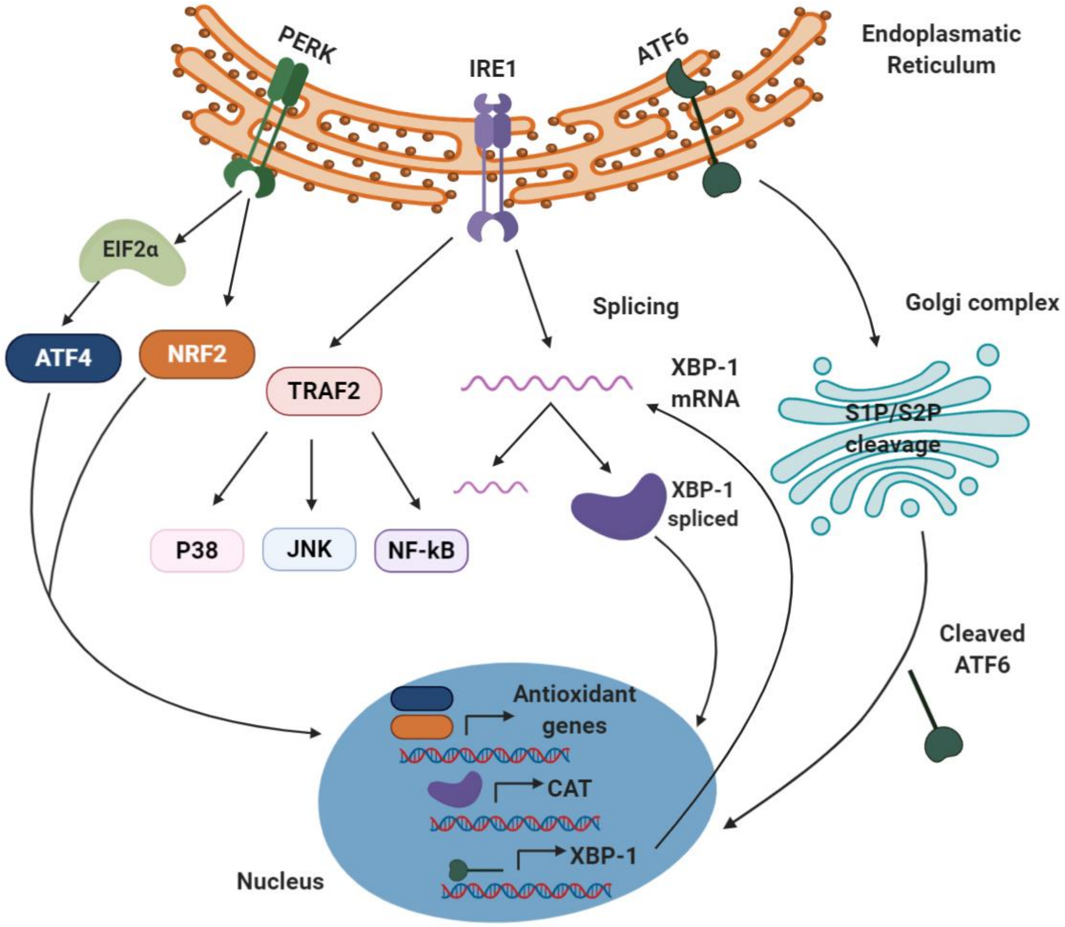

1. Liu, Y.; Adachi, M.; Zhao, S.; Hareyama, M.; Koong, A.C.; Luo, D.; Rando, T.A.; Imai, K.; Shinomura, Y. Preventing oxidative stress: A new role for XBP1. *Cell Death Differ.* **2009**, *16*, 847–857.
2. Sisinni, L.; Pietrafesa, M.; Lepore, S.; Maddalena, F.; Condelli, V.; Esposito, F.; Landriscina, M. Endoplasmic reticulum stress and unfolded protein response in breast cancer: The balance between apoptosis and autophagy and its role in drug resistance. *Int. J. Mol. Sci.* **2019**, *20*.
3. Cullinan, S.B.; Zhang, D.; Hannink, M.; Arvisais, E.; Kaufman, R.J.; Diehl, J.A. Nrf2 Is a Direct PERK Substrate and Effector of PERK-Dependent Cell Survival. *Mol. Cell. Biol.* **2003**, *23*, 7198–7209.
4. Avril, T.; Vauléon, E.; Chevet, E. Endoplasmic reticulum stress signaling and chemotherapy resistance in solid cancers. *Oncogenesis* **2017**, *6*, e373–e373.
